# What factors are associated with the increase of anemia in Ethiopian children aged 6 to 59 months between 2011 and 2016?

**DOI:** 10.1186/s40795-020-00371-6

**Published:** 2020-10-12

**Authors:** Nebyu Daniel Amaha

**Affiliations:** Department of Nutrition and Dietetics, College of Health Sciences, Mekelle University, P.O Box: 1871, Mekelle, Ethiopia

**Keywords:** Anemia, 6–59 months, Preschool children, Demographic health survey, Benishangul Gumuz, Ethiopia

## Abstract

**Background:**

In Ethiopia more than half of the children under 5 years are anemic and between 2011 and 2016 anemia in children under-5 increased by 28.7%. This study aimed to analyze this increase in anemia by socio-demographic characteristics.

**Method:**

This study was a secondary analysis of the data from the Ethiopian Demographic Health Surveys (EDHS) for 2011 and 2016. The increase of anemia was calculated using percentage change. The chi-square test was used to determine the association between anemia and six predictor variables of age, sex, mother’s educational level, residence, wealth quintile and region. The strength of association was measured using Cramer’s V.

**Results:**

Anemia increased in all age groups, both sexes, urban and rural residencies, across all wealth quintiles, all maternal education levels and all regions of Ethiopia except Benishangul Gumuz. The highest increase of anemia was seen among children born to mothers with above secondary education (65.8%), living in urban areas (40.1%), in the lowest wealth quintile (41.5%), and children from Tigray region (42.9%). Severe anemia increased in all age categories except in infants aged 9–11 months. Children of mothers with above secondary educational level had the highest increase of mild and moderate anemia. Severe anemia decreased in the second and middle wealth quintiles whereas it increased in the rest quintiles. Benishangul Gumuz is the only region where mild, moderate, and severe anemia decreased. Pearson’s chi-square (χ^2^) test showed that all the predictor variables except sex were significantly associated with anemia. Although highly significant (*p* < 0.001) using the chi-square test, Cramer’s V showed that residence (V = 0.052–0.066) and maternal education (V = 0.041–0.044) were only weakly associated with anemia.

**Conclusion:**

Anemia in children under-5 continues to be a severe public health problem in Ethiopia. Benishangul Gumuz region is the only region that was able to reduce the prevalence of all forms of anemia under-5 between 2011 and 2016, consequently other regions of Ethiopia could learn from this experience. The high increase of anemia in children born to mothers with above secondary education and highest wealth quintiles points to poor dietary practices, therefore, community based nutrition education for mothers needs to be strengthened.

## Background

Anemia affects a third of the world population and it is estimated that half of the anemia cases are caused by iron deficiency [1–4]. In developing countries, most of iron deficiency anemia (IDA) results from insufficient dietary intake and blood loss due to intestinal worm infestation [2, 5]. Dietary iron is available as either heme or non-heme form in our diets. The heme form is present in animal meat, poultry and fish and is absorbed 15–35% whereas the non-heme iron present in plant foods and dairy products is absorbed less than 10% and thus populations whose diets mainly consist of plants are at risk of iron deficiency [4–7]. Preschool children and women have the highest risk of developing anemia than other members of society [1, 8]. Adverse effects of iron deficiency in children include a decrease in cognitive, behavioral and physical growth, reduced school performance, lowered immunity and increased morbidity [6].

According to the World Health Organization (WHO) criteria [1], anemia is a severe public health problem in Ethiopia where 56.9% of children under-5 are anemic [1, 9]. Ethiopia located in the northeastern part of Africa is the most populous landlocked country in the world, and with its 112 million population the second most populous in Africa [10]. The majority of the Ethiopian population (84%) lives in rural areas and subsistence agriculture is the mainstay of livelihood and economic productivity. Ethiopia had the 7th highest burden of malnutrition in 2011 and it ranked 126th out of 156 countries in its progress towards achieving the SDG goals [11]. Previous analyses of the Ethiopian Demographic Health Survey (EDHS) found age, sex, maternal education, wealth index, region, source of drinking water, maternal body mass index (BMI) to be significant predictors of anemia in children under-5 [12–14].

Anemia in children under-5 has specifically been addressed by the Ethiopian National Nutrition Program (NPP) in its second strategic objective, which aims to improve the nutritional status of infants, young children and children under 5. The NNP-I (2008–2015) aimed to reduce anemia in children under-5 from 44 to 25% and the NNP-II (2015–2020) from 39 to 24% by 2020. Some of the initiatives include identifying and treating anemia, deworming children 2–5 years old biannually and providing micronutrient supplements to children 6–59 months. Although food fortification has been described as a cheaper and sustainable way of addressing micronutrient deficiencies in Ethiopia, Ethiopia is yet to develop and adopt national iron fortification standards [15, 16]. Despite the commitments and initiatives of the government, anemia under-5 has increased by 28.7% between 2011 and 2016 [9, 17]. This study aims to disaggregate this increase of anemia by age, sex, residence, educational level, regions, and wealth quintile; identify worse hit age groups and regions; and bring attention to the problem of anemia in children under-5 in Ethiopia.

## Methodology

### Study setting and sampling

This study used data from Demographic and Health Surveys (DHS) which are nationally-representative household surveys that provide data for a wide range of monitoring and impact evaluation indicators in the areas of population, health, and nutrition for over 90 countries since 1984. The DHS uses a stratified, two-stage cluster sampling technique. In the first stage enumeration areas (EAs) were selected from the national surveys. On second stage households within the enumeration areas were randomly selected. In EDHS 2011, 9800 children under-5 and in EDHS 2016, 9267 children participated [9, 17].

### Anemia testing and diagnosis

The EDHS program collected blood specimens for anemia testing from all children age 6–59 months for whom consent was obtained from their parents or other adults responsible for them. Blood samples were drawn from a drop of blood taken from a finger prick (or a heel prick in the case of children age 6–11 months) and collected in a microcuvette. Hemoglobin analysis was carried out on-site using a battery-operated portable HemoCue analyzer. Hemoglobin concentration differs by age, sex and physiological and pathological states and thus the cut off points for anemia in children is below 11 g/dL. Anemia was classified into three categories mild (10.0–10.9 g/dL), moderate (7.0–9.9 g/dL) and severe (less than 7.0 g/dL) [9, 17]. Hemoglobin levels were adjusted by the DHS for smoking and areas with altitudes above 1,000 m [17].

### Household wealth index

The DHS classifies households into different wealth quintiles based on the number and kinds of consumer goods they own, ranging from a television to a bicycle or car, in addition to housing characteristics such as the source of drinking water, toilet facilities, and flooring materials. These scores are derived using principal component analysis. National wealth quintiles are compiled by assigning the household score to each usual (de jure) household member, ranking each person in the household population by her or his score, and then dividing the distribution into five equal categories, each comprising 20% of the population [9, 17].

### Statistical analysis and software

The percent increase of anemia was calculated by taking the percentage difference between two survey years and dividing this value by the original (baseline) percentage value. Pearson’s chi-square was used to test for association between the six independent variables and anemia. Because the chi-square test does not indicate the strength (effect size) of a statistically significant relationship, Cramer’s V (V) was used. Cramer’s V is calculated by dividing chi-square (χ^2^) value by sample size and taking the square root of this value. The values of V range from 0 to 1, values > 0.25 are very strong; 0.15–0.25 are strong; 0.10–0.15 are moderate; 0.05–0.10 are weak and 0–0.05 are interpreted as having no association or very weak association [18]. All statistical analyses were performed using SPSS® IBM version 23.

## Results

Results in Table 1 show the prevalence of anemia in children under-5 in Ethiopia increased by 28.7%. Anemia prevalence increased in all age groups, both sexes, urban and rural residence, across all educational levels of mothers, wealth quintiles, and all regions of Ethiopia except Benishangul Gumuz region (Table 1). The highest increase was among 36–47 month infants (42.5%), males (29.4%), urban children (40.1%), women with above secondary level education (65.8%), children from lowest wealth quintile (41.5%) and children in Tigray region (42.9%).

**Table 1:** Prevalence and the percentage increase of anemia by socio-demographic factors in Ethiopia between 2011 and 2016

Characteristic		2011 (*n* = 9800)		2016 (*n* = 9267)		% increase
		Frequency	%	Frequency	%	
Age in months	6–8	351	61.3	428	78.0	27.2
	9–11	364	72.7	377	76.3	4.9
	12–17	624	62.6	815	72.1	15.2
	18–23	470	52.2	584	65.5	25.5
	24–35	936	45.4	1149	59.0	29.9
	36–47	871	35.8	1030	51.0	42.5
	48–59	719	30.8	894	40.0	29.9
Sex of child	Male	2229	44.3	2757	57.3	29.4
	Female	2107	44.2	2522	56.6	28.1
Residence	Urban	401	35.2	462	49.3	40.1
	Rural	3932	45.4	4815	57.8	27.3
Mother’s Education	No education	2978	45.5	3442	58.2	27.9
	Primary	1049	42.9	1331	56.8	32.4
	Secondary	72	41.4	172	48.8	17.9
	Above Secondary	37	30.1	88	49.9	65.8
Wealth quintile	Lowest	1061	47.9	1467	67.8	41.5
	Second	1038	47.6	1248	57.6	21.0
	Middle	895	43.3	1033	52.6	21.4
	Fourth	856	43.1	930	54.0	25.3
	Highest	486	35.9	599	47.9	33.4
Region	Tigray	248	37.5	328	53.6	42.9
	Affar	71	74.7	68	74.8	0.1
	Amhara	754	35.1	785	42.2	20.2
	Oromiya	2171	51.7	2625	65.5	26.7
	Somali	166	68.7	308	82.9	20.7
	Ben-Gum*	52	46.5	41	42.5	−8.6
	SNNPR*	779	36.9	996	50.0	35.5
	Gambela	15	50.9	12	56.2	10.4
	Harari	11	55.5	11	67.9	22.3
	Dire Dawa	19	33.2	25	49.2	25.9
	Addis Ababa	51	62.9	81	71.5	25.4
Total			44.2		56.9	28.7

Calculation of % increase = (% in 2016 - % in 2011) / (% in 2011); * *Ben-Gum* Benishangul Gumuz region; *SNNP South Nations and Nationalities People’s region

All variables except sex were significantly associated with anemia in both survey years (Table 2). There was a strong association between anemia and age category of a child (*p* < 0.0001, Crammer’s V > 0.25). Although highly significant (*p* < 0.001), there was a weak strength of association between the anemia status of a child and residence (V = 0.052–0.066), maternal education (V = 0.041–0.044). The relationship between wealth quintile and anemia increased from weak association (V = 0.079) in 2011 to moderate association (V = 0.134) in 2016, and the association between regions of Ethiopia and anemia status increased from moderate (V = 0.185) to strong (V = 0.222) association (Table 2).

**Table 2:** Socio-demographic factors assoicated with anemia in Ethiopian children under-5 between 2011 and 2016, Ethiopian Demorgaphic Health Survey

Characteristic		2011			2016		
		χ^2^	*p-value*	V	χ^2^	*p-value*	V
Age in months	6–8	634.7	0.001	0.254	601.6	0.001	0.255
	9–11						
	12–17						
	18–23						
	24–35						
	36–47						
	48–59						
Sex	Male	0.011	0.919	0.001	0.457	0.52	0.007
	Female						
Residence	Urban	42.4	0.001	0.066	24.8	0.001	0.052
	Rural						
Mother’s Education	No education	15.9	0.001	0.041	16.9	0.001	0.044
	Primary						
	Secondary						
	Above Secondary						
Wealth quintile	Lowest	61.6	0.001	0.079	166.9	0.001	0.134
	Second						
	Middle						
	Fourth						
	Highest						
Region	Tigray	335.7	0.001	0.185	457.6	0.001	0.222
	Affar						
	Amhara						
	Oromiya						
	Somali						
	Ben-Gum*						
	SNNP*						
	Gambela						
	Harari						
	Dire Dawa						
	Addis Ababa						

χ^2^ = chi-square test; *p*-value of chi-square test; V=Cramer’s V; *Ben-Gum Benishangul Gumuz region; *SNNP South Nations and Nationalities People’s region.

**Fig. 1:**
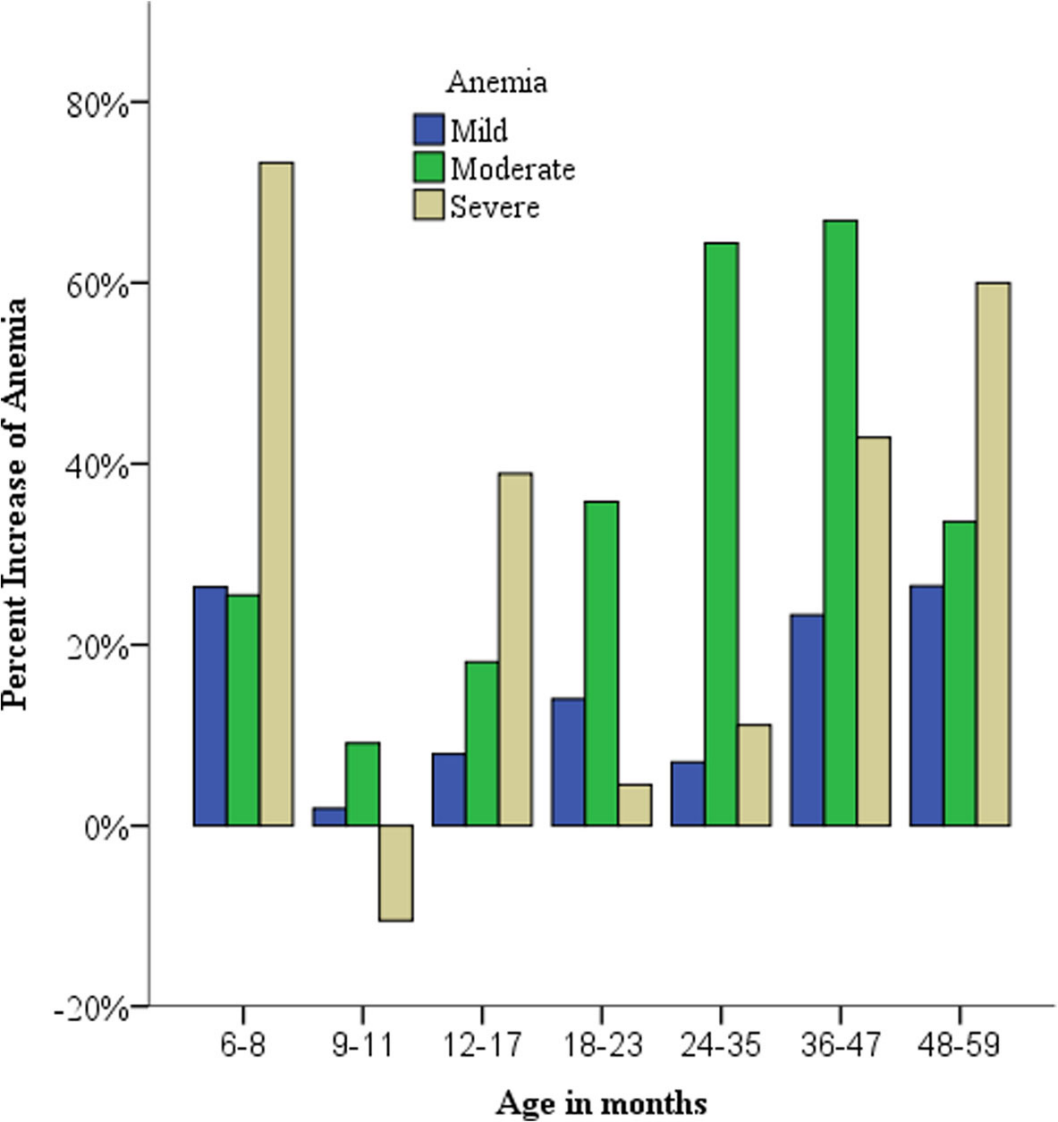
Increase of anemia in Ethiopian children under-5 by age in months between 2011 and 2016

**Fig. 2:**
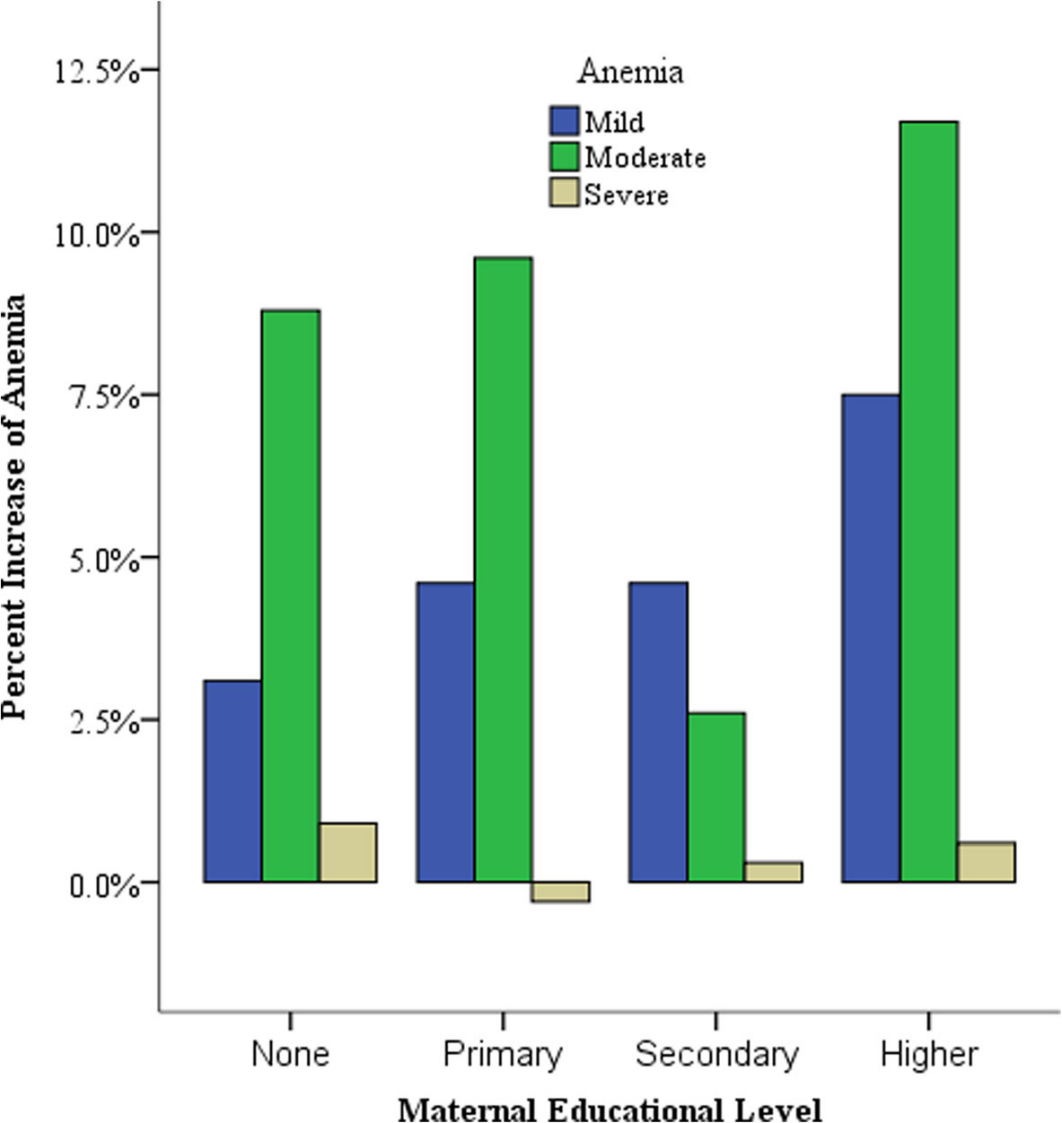
Increase of anemia in children under-5 in Ethiopia by maternal educational levels

**Fig. 3:**
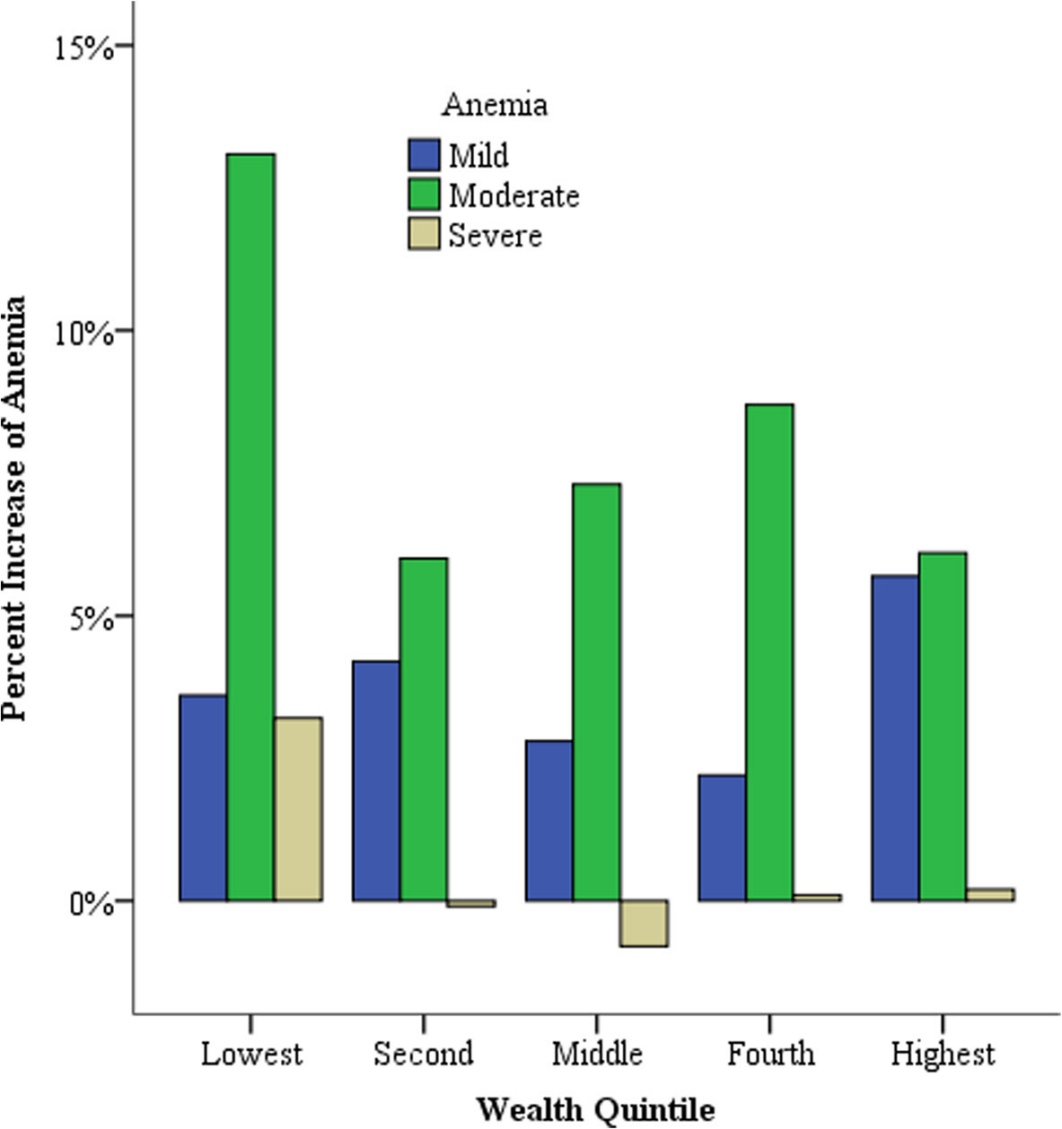
Increase of anemia in Ethiopian children under-5 by household wealth quintiles

**Fig. 4:**
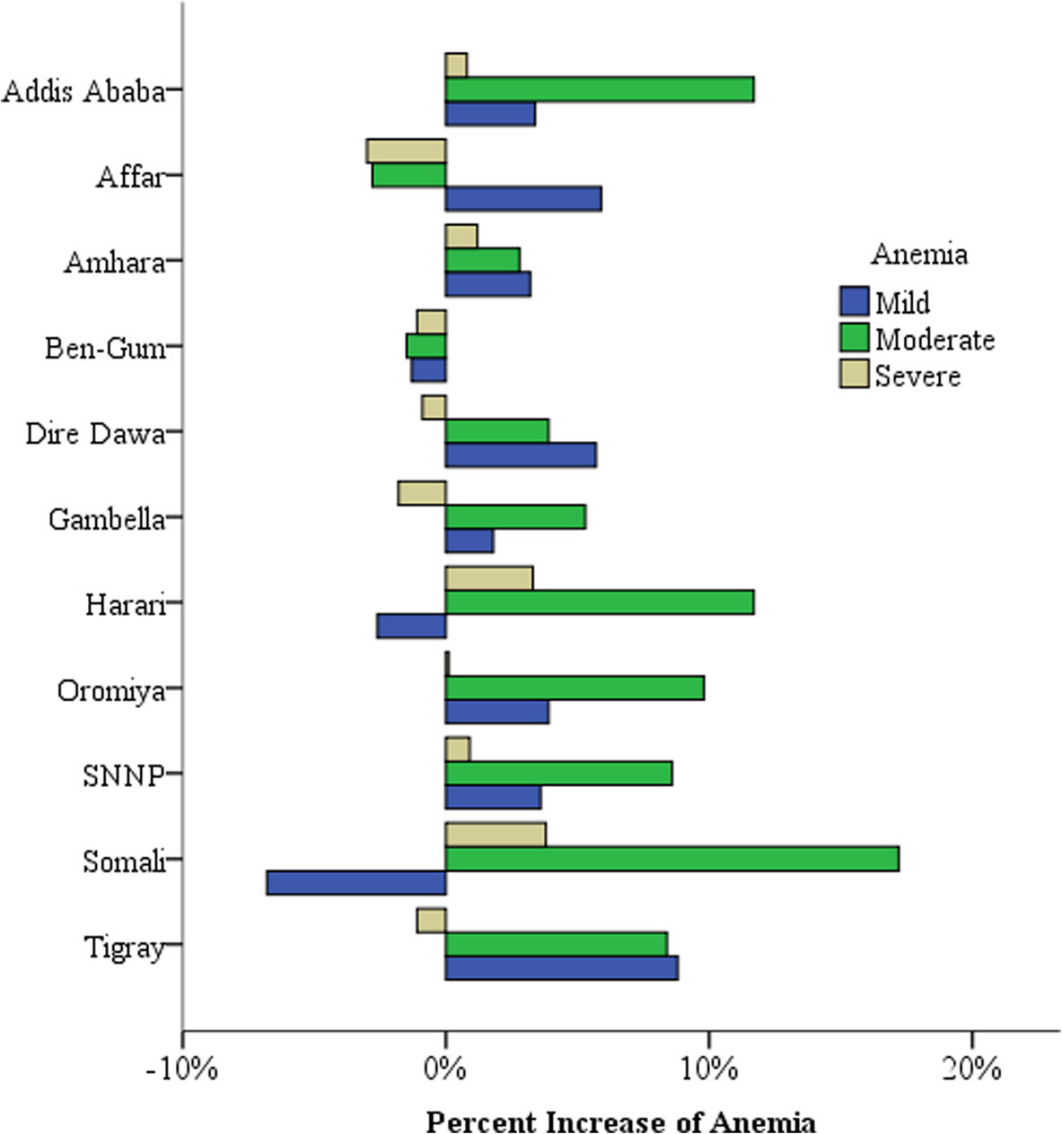
Regional differences in the increase of anemia in Ethiopian children under-5 between 2011 and 2016. Ben-Gum = Benishangul Gumuz region; SNNP=Southern Nations Nationalities People’s region

Figure 1 shows that severe anemia decreased in the 9–11 month age category and the highest increase of severe anemia was among children 6–8 months (Fig. 1). The highest increase of mild and moderate anemia was among children born to mothers with above secondary educational level whereas severe anemia decreased among mothers with primary level of education (Fig. 2). Children born in the lowest wealth quintile had the highest increase of moderate and severe anemia. Severe anemia decreased in children born from the middle wealth quintiles and the highest increase of mild anemia was in the highest wealth quintile (Fig. 3). Benishangul Gumuz is the only region where all forms of anemia declined. The Somali region had the highest increase of moderate and severe anemia, at the same time the highest decrease of mild anemia (Fig. 4).

## Discussion

This study analyzed the increase of anemia in children under-5 in Ethiopia between 2011 and 2016. Further analysis of this increase in anemia is required to understand which socio-demographic segments were more affected. More than half, 56.9%, of Ethiopian children under-5 are anemic according to the 2016 EDHS. This is a little lower than the average anemia prevalence of 59.9% in children under-5 from 27 Sub-Saharan African countries [19]. Anemia in children is a severe public health problem in Ethiopia and needs urgent attention to address its underlying causes.

Anemia increased across all age categories and children aged 36–47 months had the highest increase (42.5%) of anemia (Table 1). Age had the highest (Cramer’s V = 0.25) strength of association with anemia than the rest of the predictor variables studied (Table 2). Similar to findings from Armenia [20] and India [21], infants aged 9–11 months had the highest prevalence (72.7%) of anemia. At the same time except for 9–11 month infants, all forms of anemia increased across all age categories (Fig. 1). The lowest affected group was 48–59 months, this is a time range when the child’s growth rate decreases, a better food variety is available for the child, and the child is physiologically more equipped to take part in the family dining table, for example mastication of meat [22]. The highest increase of severe anemia was among children 6–8 months (Fig. 1). The increase of anemia after reaching 6 months could be due to the depletion of fetal iron stores [6] and the increased iron requirements placed on the body around 6 months of exclusive breastfeeding after birth [1, 23].

Regardless of their educational level, anemia increased in children born to mothers of all educational levels. The highest prevalence of anemia is among children born from mothers with no formal education (Table 1). The highest increase in anemia (65.8%) was seen in children born to mothers with higher than secondary education (Table 1). Although the smaller sample size and lower baseline prevalence of anemia could be a possible reason, this finding is counter-intuitive because mothers with higher levels of education are more likely to be employed and have better knowledge about infant feeding. It’s important to note that having an above secondary level education does not necessarily mean the mothers are employed. Moreover, educated mothers could be practicing poor dietary habits themselves and for their children because they spend many hours at work. Between 2011 and 2016, the gap in the prevalence of anemia between children born to mothers with no education and above secondary education has narrowed from 15 to 8% (Table 1). Figure 2 shows that mild and moderate anemia showed the highest increase among mothers above secondary level education (Fig. 2).

Anemia increased across all the wealth quintile groups and the highest increase (41.5%) was among children born from the lowest wealth quintile (Table 1). Severe anemia decreased among children born in the second and middle wealth quintile (Fig. 3). Household wealth index was significantly associated with the anemia status of children (*p* < 0.0001). This association is supported by some studies [13, 24, 25], however, other studies in Ethiopia [14, 26, 27] have reported that there was no statistically significant relationship between household wealth and anemia. The strength of association between anemia and wealth quintile increased from weak (χ^2^ = 61.6, V = 0.079) in 2011 to moderate (χ^2^ = 166.9, V = 0.134), moreover, the gap between the highest and lowest quintiles increased from 12% in 2011 to 20% in 2016 (Table 1). The highest wealth quintile showed the second highest increase (33.4%) of anemia (Table 1). This increase in the highest wealth quintile is unexpected because families with the highest income are more likely to be food secure and provide better healthcare to their children. Food secure households might lack adequate dietary practices and engaged in eating less nutritious, undiversified foods. Furthermore, the highest quintile doesn’t necessarily mean they are rich; it simply represents the highest quintile in comparison with the rest of the population. Furthermore, most of the increase of anemia in the highest wealth quintile is mostly mild anemia (Fig. 3).

Anemia in children shows significant differences across Ethiopian regions. Except for Benishangul Gumuz region, anemia increased in all the regions of Ethiopia (Table 1). The highest increase was in Tigray (42.9%) and South Nations Nationalities People’s (SNNP) regions (35.5%). Somali region had the highest decrease in mild anemia and the highest increase in moderate anemia from its baseline in 2011. Moderate and mild anemia decreased in Affar and Benishangul Gumuz regions but Benishangul Gumuz is the only region where all forms of anemia declined (Fig. 4). Because regional administrations in Ethiopia are ethnically based, anemia might be affected by cultural and dietary practices of populations living in a given region.

Regional health and nutrition reports from Benishangul Gumuz exhibit indicators which are better than most regions and possibly explain why anemia could have decreased in this region. For example, Benishangul Gumuz has the second-highest minimum dietary diversity score (eating from at least four food groups) and the second-highest minimum acceptable diet when compared with the other regions. Additionally, Benishangul Gumuz has the second-highest median breastfeeding duration, the lowest prevalence of severe anemia and the second lowest prevalence of anemia in children. It also has the lowest percentage of children who described as “very small” after birth and the lowest percentage of children under-5 with fever in the 2 weeks before the survey. When compared with normal practice, children in Benishangul Gumuz receive the highest increase in food during diarrhea [9]. A national nutrition survey in 2015 reported that Benishangul Gumuz had the second lowest vitamin A deficiency after the capital, Addis Ababa, and the highest percentage of children who drunk thin porridge (semisolid food) before the survey [28]. Thus, the decrease in anemia in children in Benishangul Gumuz could be partly due to the dietary and childcare practices of the people living in this region.

Ethiopia is off course in meeting its objective of decreasing anemia in children under-5 as outlined in its NNPs. The increase of anemia attests that the implementation of the nutrition programs needs to be strengthened and coordinated more efficiently. The other regions of Ethiopia could benefit from the successful experience of Benishangul Gumuz in reducing anemia in children. Further studies regarding infant feeding practices and dietary customs in Benishangul Gumuz region are required to identify the reasons behind this exceptional decrease of anemia in this region. Because this study was a secondary analysis of a cross-sectional study, it is associated with the cause-effect problem of such cross-sectional studies. Hemoglobin was the only indicator used to measure the anemia status of children, thus the specific type of anemia could not be determined.

## Conclusion

The increase of anemia in children under-5 was in all age groups, both sexes, in urban and rural residences, across all maternal educational levels and wealth quintiles. Except for Benishangul Gumuz region, anemia increased in all regions of Ethiopia. Because infants 6–8 months had the highest increase of severe anemia, interventions regarding complementary feeding ought to be prioritized. The protective effects of maternal education and household wealth were less significant high increases of mild and moderate anemia were also observed in women with above secondary level education and the highest wealth quintiles. Therefore, comprehensive community-based nutrition education in Ethiopia should be strengthened.

## Data Availability

The data that support the findings of this study are available from the DHS website at https://dhsprogram.com/where-we-work/Country-Main.cfm?ctry_id=65&c=Ethiopia&r=1.

## Abbreviations

BMI: Body Mass Index
Ben-Gum: Benishangul Gumuz region
IDA: Iron Deficiency Anemia
WHO: World Health Organization
DHS: Demographic Health Survey
EDHS: Ethiopian Demographic Health Survey
NNP: National Nutrition Program
SNNP: Southern Nations Nationalities People’s region
